# Analysis of the Transcriptional Program of Developing Induced Regulatory T Cells

**DOI:** 10.1371/journal.pone.0016913

**Published:** 2011-02-09

**Authors:** Iryna Prots, Alla Skapenko, Peter E. Lipsky, Hendrik Schulze-Koops

**Affiliations:** 1 Division of Rheumatology, Medizinische Poliklinik, University of Munich, Munich, Germany; 2 National Institute of Arthritis and Musculoskeletal and Skin, National Institutes of Health, Bethesda, Maryland, United States of America; University of Nebraska Medical center, United States of America

## Abstract

CD25+ regulatory T cells develop in the thymus (nTregs), but may also be generated in the periphery upon stimulation of naive CD4 T cells under appropriate conditions (iTregs). To gain insight into the mechanisms governing iTreg development, we performed longitudinal transcriptional profiling of CD25+ T cells during their differentiation from uncommitted naive CD4 T cells. Microarray analysis of mRNA from CD25+ iTregs early after stimulation revealed expression of genes involved in cell cycle progression and T cell activation, which largely overlapped with genes expressed in CD25+ effector T cells (Teffs) used as a control. Whereas expression of these genes remained elevated in Teffs, it declined gradually in developing iTregs, resulting in a more quiescent phenotype in mature iTregs. A similar pattern of kinetics was observed for biological processes and for intracellular pathways over-represented within the expressed genes. A maximum dichotomy of transcriptional activity between iTregs and Teffs was reached at late stages of their maturation. Of interest, members of the FoxO and FoxM1 transcription factor family pathways exhibited a reciprocal expression pattern in iTregs and Teffs, suggesting a role of these transcription factors in determining T cell fate.

## Introduction

CD25+ regulatory T cells (Tregs) are a specialized subset of CD4 T cells. Tregs play a crucial role in establishing and maintaining peripheral self-tolerance and in terminating immune reactions by suppressing the activity of effector T cells (Teffs) and other immune cells [1]–[3]. They are characterized by the expression of the forkhead box P3 (Foxp3) transcription factor and constitute 5–10% of the peripheral CD4 T cell pool [4]. Deficiencies in Foxp3 lead to severe systemic autoimmunity, and compromised development and/or function of Tregs is associated with the development of autoimmune diseases [5]–[9]. Moreover, reconstitution of Tregs ameliorates disease activity in several animal models of autoimmunity, inflammation, and graft rejection [10]–[14], indicating a promising therapeutic potential of Tregs and consequently the necessity to understand in detail their development and function.

Tregs were initially found to be generated during T cell development in the thymus (natural occurring Tregs; nTregs) [15]. However, it has now become clear that Tregs can also be generated from naive CD4 T cells in peripheral lymphoid tissues (induced Tregs; iTregs) and that peripheral Treg development might represent a significant source of circulating Tregs [16]–[18]. Prolonged exposure to peripheral antigens or suboptimal costimulation during antigen presentation has been described to initiate the development of iTregs [19]. Different soluble factors, such as cytokines, retinoic acid or neuropeptides provide additional signals, further facilitating Foxp3 upregulation and the generation of peripheral Tregs [20]–[22]. We have demonstrated that suboptimal activation of naive CD25- CD4 T cells in the presence of IL-4 induces the generation of functionally competent Foxp3+ iTregs [23].

Although Foxp3 induction and Foxp3-orchestrated expression of a number of Treg-specific molecules, such as CD25, cytotoxic T-lymphocyte antigen 4 (CTLA4), glucocorticoid-induced tumor necrosis factor receptor (GITR) and CD127, are thought to play a central role in Treg differentiation [24]–[26], a meta-analysis of Treg-transcriptional signatures strongly suggested the involvement of additional regulatory elements [27]. To gain insight into the molecular program of extrathymic Treg development, we analyzed the global gene expression profile of CD25+ Tregs generated *in vitro* from peripheral naive CD25- CD4 T cells in the presence of autologous feeder cells and IL-4. At early developmental stages (days 3 and 5), iTreg development was characterized by a highly active gene expression status that was not overtly different than that of developing Teffs, as most of the genes expressed at that time represented biological processes and pathways involved in proliferation and cell cycle progression. With prolonged development, the transcriptional program of iTregs diminished steadily, resulting in about three times lower numbers of genes expressed in iTregs as compared to Teffs at day 10, whereas the gene diversity between the two populations achieved its maximum. Two pathways of the Fox transcription factor family, “FoxO family” and “FoxM1 transcription factors”, were identified to be specifically over-represented during the development in iTregs and Teffs, respectively, and might, therefore, represent decisive molecular pathways specifying iTreg development and activation of Teffs, respectively, providing additional insight into the transcriptional programs potentially involved in iTreg development.

## Materials and Methods

### Reagents and Abs

The following mAbs and reagents were used for purification, stimulation, and staining of human cells: anti-CD16 (3g8FcIII), anti-CD3 (OKT3), anti-CD8 (OKT8), anti-CD45RO (UCHL-1), and anti-HLA-DR (L243; American Type Culture Collection, Manassas, VA); anti-CD19 (Dako Cytomation, Glostrup, Denmark); FITC-conjugated anti-CD3, PE-labeled anti-CD4, and FITC-labeled anti-CD4 (Sigma-Aldrich, Taufkirchen, Germany); PE-labeled anti-CD25, FITC-labeled anti-CD27, FITC-labeled anti-CD45RA, and PE-labeled anti-CD45RO (BD Bioscience, Heidelberg, Germany); polyclonal goat anti-mouse immunoglobulin (Ig) (MP Biomedicals, Solon, OH); sheep red blood cells (SBRC) (Fiebig-Nährstofftechnik, Idstein, Germany), fetal calf serum (FCS), phosphate-buffered saline (PBS) (Life Technologies, Carlsbad, CA), normal human serum (NHS). Human recombinant IL-4 was obtained from Endogen, Rockford, IL.

### Cell purification

Peripheral blood mononuclear cells (PBMC) were obtained from heparinized venous blood donated by healthy individuals by centrifugation over a Ficoll-Hypaque gradient (Sigma-Aldrich). For isolation of T cells, PBMC were incubated with SRBC as described previously [28]. The rosette-negative cells were used as T cell-depleted PBMC (feeder cells). The rosette-positive cells were further purified by negative selection panning with mAbs to CD8, CD16, CD19, HLA-DR, and CD45RO as described previously [29]. CD25+ and CD25- CD4 cell populations were isolated from the naive CD4 T cells using CD25 magnetic microbeads from Miltenyi Biotec (Bergisch Gladbach, Germany) according to the manufacturer's instructions. The homogeneity and purity of all isolated populations were routinely controlled by flow cytometry. Typically, ≥95% of the cells were positive for CD3 and CD4 and ≥95% of the isolated naive cells were positive for CD45RA and negative for CD45RO. Naive CD25- CD4 T cells were ≥98% negative for CD25, while CD25+ cells were ≥90% positive for CD25. More than 98% of the cells were viable after purification. The study was approved by the ethics committee of the University of Erlangen, and all subjects gave their written informed consent.

### Flow cytometry

For surface staining, T cells (1×10^5^/sample) were washed with PBS containing 2% FCS, incubated with saturating amounts of directly fluorochrome-labeled mAb against diverse surface molecules for 15 minutes at 4°C, washed, and analyzed by flow cytometry (Cytomics FC500; Beckman Coulter, Fullerton, CA).

### Generation of CD25+ iTregs

All cell cultures were conducted in RPMI 1640 medium supplemented with penicillin G/streptomycin (50 U/ml), L-glutamine (2 mM; all from Life Technologies), and 10% NHS at 37°C in a humidified atmosphere containing 5% CO_2_. CD25+ iTregs were generated *in vitro* as previously described [23]. Briefly, purified naive CD25- CD4 T cells were incubated at a concentration of 1×10^6^/ml in the presence of 1×10^6^/ml irradiated (30 Gy) autologous feeder cells in a final volume of 2 ml in 24-well cell culture plates (Costar, Cambrige, MA) for 3, 5, 7 and 10 days. IL-4 at a final concentration of 6.25 ng/ml was added to the cultures (Fig. S1A). Control cultures were incubated without exogenous IL-4 and resulted in the generation of activated effector T cells. At indicated time points, cells were harvested, counted, analyzed for surface expression of CD4 and CD25, and processed for CD25+ and CD25- T cell magnetic isolation.

### Proliferation assay

CD25+ and CD25- T cells recovered after a 10-day culture (50×10^3^/well) were cultured together (at a 1:1 ratio) or separately in triplicates for 3 days in 96-well, round-bottom plates (Costar) in the presence of soluble anti-CD3 mAb (1 µg/ml) and in the presence of 100×10^3^ autologous irradiated feeder cells. Incorporation of [^3^H]TdR (1 µCi/well) by proliferating lymphocytes during the last 16 h of the culture was measured using a liquid scintillation counter on a 1205 Wallac Betacounter (Wallac/Pharmacia, Turku, Finnland).

### Preparation of total RNA and real time PCR

Total RNA was extracted from freshly isolated or cultured CD25+ and CD25- T cells at indicated time points using the RNeasy Minikit (Qiagen, Hilden, Germany) with an additional DNA digestion step (RNase Free DNase Set; Qiagen). 0.1–1 µg of total RNA was transcribed to cDNA for 1 h at 42°C in a total volume of 20–50 µl containing 1× avian myoblastosis virus reverse transcriptase (AMV RT) buffer (Promega, Mannheim, Germany), 1 mM dNTPs, 100 ng/ml oligo(dT)_12–18_ (all from GE Healthcare, Munich, Germany), and 0.25 U/µl AMV RT (Promega). Real time PCR was performed in duplicate in a final volume of 25 µl using either 1× the Universal PCR Master Mix and 1× TaqMan Gene Expression Assays-on-Demand for Foxp3, FoxO3a, FoxM1 and cyclophilin A (all from Applied Biosystems, Darmstadt, Germany) or 1× Power SYBR Green PCR Master Mix (Applied Biosystems) and 70.4 nM primer mix for SYBR Green detection for MAFF, CCR2, PGDS, PMCH and EF1A1 (MWG-Biotech, Ebersberg, Germany) in the ABI PRISM 7000 Sequence Detection System (Applied Biosystems). Primers for SYBR Green detection were designed using the MacVector software (Accelrys, Cambridge, UK) to amplify fragments spanning exon-exon junctions of up to 120 bp in length. Forward-reverse primer pairs for SYBR Green PCR were as follows, respectively: 5′-CCAGCAAAGCTCTAAAGATCAAGC-3′ and 5′-AGATGCCGGTTCAGCTCG-3′ for MAFF; 5′-CGTTGGGGAGAAGTTCAGAAGC-3′ and 5′-TTTTTGGAGTGGGGCAATCC-3′ for CCR2; 5′-TGGTAACTCTGTAACTTGGGCAGAC-3′ and 5′-GGATGGTTGTCTAACAGGTCAGGC-3′ for PGDS; 5′-GGAAGGAGAGATTTTGACATGCTC-3′ and 5′-GATGATGTGGACCAACAGGTATCAG-3′ for PMCH; 5′-GTTGATATGGTTCCTGGCAAGC-3′ and 5′-GCCAGCTCCAGCAGCCTTC-3′ for EF1A1. The PCR program was as follows: 95°C for 10 min followed by 40 cycles of 95°C for 10 sec and 60°C for 1 min and (for SYBR Green detection) 1 cycle of 95°C for 15 sec, 60°C for 30 sec and 95°C for 15 sec. Relative quantification was performed by calculating the difference in cross-threshold values (ΔCt) of the gene of interest and a housekeeping gene, cyclophilin A and/or EF1A1, according to the formula 2^-ΔCt^. Where indicated, the relative expression values were normalized to the expression values in the control condition.

### Control of total RNA quality

Total RNA quality was controlled electrophoretically using the RNA 6000 Pico Assay (Agilent Technologies, Santa Clara, CA) according to the manufacturer's instructions. The electrophoretic RNA separation was performed in an Agilent 2100 Bioanalyzer and visualized by the 2100 expert software (both from Agilent Technologies). A successful ladder run resulted in six well-resolved RNA peaks. The electrophoregram of a high quality total RNA sample consisted of two well-separated ribosomal peaks.

### Two-step biotion-labeled cRNA synthesis and microarray hybridization

100 ng of total RNA were used for synthesis of biotin-labeled cRNA for microarray hybridization by two cycles of amplification using the GeneChip Two-Cycle cDNA Synthesis Kit, GeneChip IVT Labeling Kit (both from Affymetrix, Santa Clara, CA) and the MEGAscript T7 Kit (Ambion, Austin, TX) according to the manufacturers' instructions. 20 µg of biotin-labeled cRNA were fragmented with 1× fragmentation buffer (Affymetrix) in a final volume of 40 ml at 94°C for 35 min. The size distribution of fragmented biotinylated cRNA samples was analyzed electrophoretically using the RNA 6000 Pico Assay as described above. 15 µg of fragmented cRNA were used to hybridize on the GeneChip HG_U133A array (Affymetrix). Hybridization was performed in a GeneChip Hybridization Oven 640 (Affymetrix) for 16 hours at 45°C before arrays were washed and stained on a GeneChip Fluidics Station 400 (Affymetrix), and scanned with a GeneChip Scanner 3000 (Affymetrix).

### Microarray analysis

Signal intensities of the probes on the array were imported into the microarray data analyzing software GeneSpring GX10 (Agilent Technologies) and an absolute detection call for each probe (present, marginal, or absent) was assigned by a MAS 5 probe summarization algorithm. The whole analysis of the microarray data was performed using the GeneSpring GX10 software. Baseline transformation to median of all samples was applied to each probe to normalize signal intensities of each probe across all samples to its median. Further analysis was performed with probes that had a present or marginal call in at least one out of all analyzed samples. Three independent experiments were performed for microarrays and gene expression was analyzed between sample groups consisting of three replicates, except a group of a 10-day Teffs with two replicates. The analysis was performed in several steps (Fig. S1). First, the probes with signal intensities not significantly different (p>0.05) between CD25- cells at either time point and naive CD25- CD4 T cells (day 0) were determined by the repeated measures one-way analysis of variance (ANOVA). Here and latter, the Benjamini-Hochberg correction for p-value calculation was applied to correct for multiple testing.

Probes not regulated in CD25- cells were further analyzed by a two-way ANOVA between iTregs or Teffs and the respective CD25- T cell population at all time points followed by Tukey's Honestly Significant Difference (TukeyHSD) post hoc test to identify significantly different probes at each particular time point. The fold-change analysis was than applied to identify significant probes at each time point with at least 2-fold differential expression between means of three experiments in iTregs or Teffs and the respective CD25- cell population (“iTreg-" and “Teff-regulated probes”, respectively).

iTreg-specific and Teff-specific transcripts were identified within the iTreg- and Teff-regulated probes, respectively as probes having a significant (p<0.05) at least 2-fold differential expression between iTregs and Teffs in each indipendent experiment at indicated time points as determined by one-way ANOVA followed by TukeyHSD test and the fold-change analysis. iTreg- and Teff-specific probes identified at different time points were hierarchically clustered based on their expression signals in iTregs and Teffs at the respective time point applying a Euclidean similarity measure and Complete linkage rule.

The lists of iTreg- and Teff-regulated probes at each time point were examined for a significant over-representation of biological processes (p<0.1) and for a significant overlap with human pathways (p<0.05) by Gene Ontology (GO) analysis on biological process terms and “Find Significant Pathway Analysis”, respectively. Both analyses use a hypergeometric test for a p-value computation. In case of the GO analysis, a Benjamini-Yekutieli correction was applied to account for multiple GO term testing. Over-represented biological processes were graphically visualized using Graphviz software (http://www.graphviz.org) (Fig. S3). Find Significant Pathway analysis used immune and cancer signaling pathways from the Cancer Cell Map (http://cancer.cellmap.org/cellmap) and BioCyc database (http://biocyc.org) (pre-loaded in GeneSpring GX10), human pathways from Kyoto Encyclopedia of Genes and genomes (KEGG; http://www.genome.jp/kegg) and Nature Pathway Interaction (http://pid.nci.nih.gov) databases (both imported into GeneSpring GX10). Only pathways with at least 50% representation on the microarray platform (number of pathway-related genes on the platform in relation to the total number of genes in the pathway ×100%) were included into the analysis. All microarray data are MIAME compliant and have been deposited in a MIAME compliant database, e.g. NCBI's Gene Expression Omnibus (GEO) (http://www.ncbi.nlm.nih.gov/geo) and are accessible through GEO Series accession number GSE24634.

### Statistical analysis

Chi-square test was calculated by using GraphPad Prism 5.0 software (GraphPad Software, La Jolla, CA). Expression microarray data of the candidate genes and the results of real-time PCR were analyzed by the two-tailed paired Student's *t* test. p-values ≤0.05 were considered as statistically significant differences.

## Results

### Induction of CD25+ iTregs by priming with IL-4

**Figure 1:**
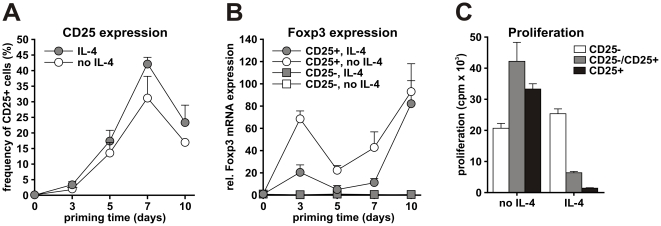
CD25+ iTreg development. Human naive CD4 T cells depleted of CD25+ cells were cultured with autologous irradiated feeder cells for 10 days in the presence or absence of IL-4. (A) At days 3, 5, 7 and 10 cells were analyzed for CD25 expression. (B) CD25+ and CD25- populations were magnetically sorted at the days indicated. Foxp3 mRNA expression was assessed in both CD25+ and CD25- populations by real-time PCR and its relative expression was calculated in relation to cyclophilin mRNA. (C) At day 10, the proliferative and suppressive capacities of CD25+ and CD25- cells in response to CD3 stimulation were assessed by thymidine incorporation. Data are shown as a mean ± SEM of three independent experiments (A, B) or as a mean ± SD of one representative experiment performed in triplicates (C).

To generate CD25+ iTregs, we cultured naive CD4 T cells for 10 days with feeder cells in the presence of IL-4 as previously described [23]. To control for successful CD25+ iTreg development, cells were analyzed during the culture for CD25 surface expression and for Foxp3 mRNA expression, and at the end of differentiation (e.g. day 10) for their proliferative and suppressive capacity. As shown in Figure 1A, increasing numbers of CD25+ T cells were detected in the differentiating cultures irrespective of the presence of IL-4. Both CD25+ T cell populations expressed Foxp3 mRNA (Fig. 1B). At day 10, however, only the CD25+ T cells purified from IL-4-containing cultures were anergic and possessed a regulatory capacity as they suppressed proliferation of CD25- cells in response to CD3 stimulation (Fig. 1C). CD25+ T cells derived in the absence of IL-4 proliferated vigorously in response to CD3 stimulation, did not inhibit CD25- T cell proliferation (Fig. 1C), produced high levels of the effector cytokines IFNγ and TNF after restimulation with PMA/ionomycine [23] and thus, represented activated effector T cells. They were used, therefore, as a non-iTreg control (CD25+ Teff) throughout the study.

### Identification of CD25+ iTreg-specific genes

To analyze the transcriptional program during CD25+ iTreg development, RNA from developing cells was analyzed at different time points of culture (day 3, 5, 7 and 10) by microarray analysis using the Affymetrix GeneChip HG_U133A (Fig. S1A). We restricted the analysis to those transcripts whose expression was not significantly altered in CD25- T cells as compared to the starting population during the entire culture period (Fig. S1B, Step 1). Of these, a total of 1,558 and 1,707 probes (representing 1,358 and 1,487 transcripts, respectively) were identified to show a more than two-fold different expression (two-way ANOVA; p<0.05) in CD25+ iTregs and in Teffs compared to the appropriate CD25- cell population in at least one analyzed time point (Fig. S1B, Step 2). Of the CD25+ T cell-regulated probes, 912 and 1,006 (representing 778 and 861 transcripts, respectively) were up-regulated in CD25+ iTregs and Teffs, respectively, in at least one time point but not down-regulated at any time point, whereas 646 and 700 probes (576 and 621 transcripts, respectively) were down-regulated in at least one time point but not up-regulated at any time point in iTregs and Teffs, respectively.

**Figure 2:**
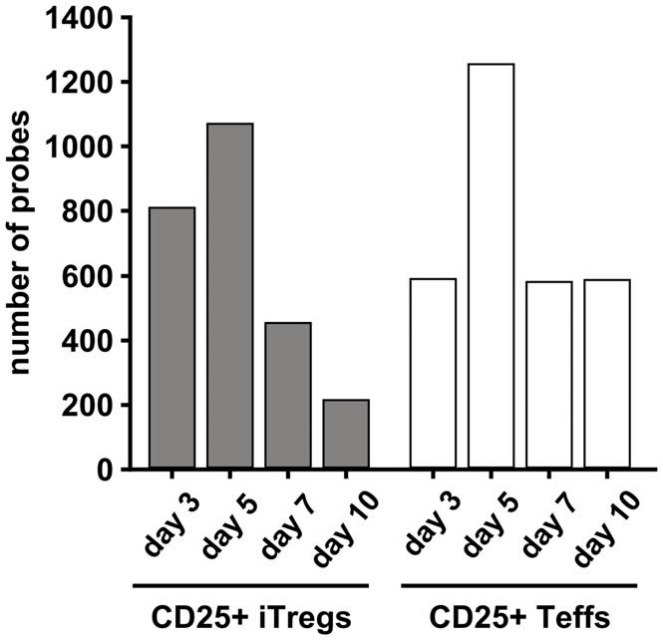
Number of regulated transcripts in CD25+ T cells during 10 days of culture. Bars indicate numbers of probes with at least two-fold differential expression between CD25+ iTregs or Teffs and their corresponding CD25- cell population at each analyzed time point.

The pattern of the expression of transcripts regulated in CD25+ T cells during the culture period was different between CD25+ iTregs and Teffs (p<0.0001) (Fig. 2). After a peak at day 5, the numbers of up- or down-regulated probes decreased steadily through day 7 to day 10 in CD25+ iTregs but remained at a similar level at days 7 and 10 in CD25+ Teffs. The number of regulated probes at day 10 in CD25+ Teffs was almost three times that of CD25+ iTregs (580 vs 209, respectively; Fig. 2).

**Figure 3:**
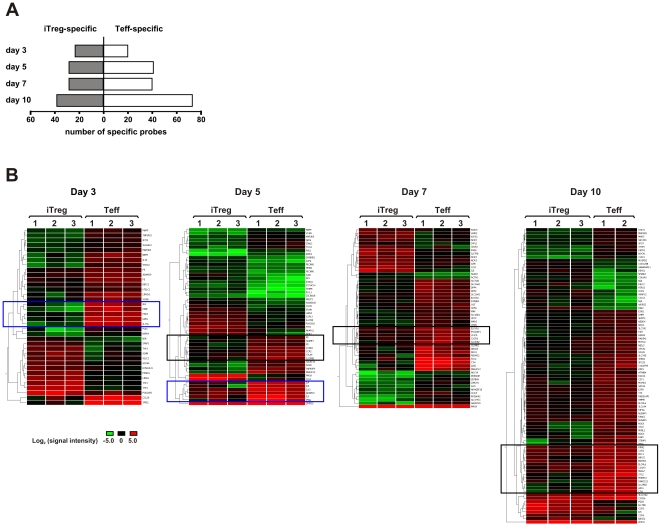
iTreg and Teff specific transcripts during development. (A) Number of probes specifically expressed in iTregs and Teffs at different times during differentiation. (B) Hierarchical clustering was performed on 93 iTreg-specific and 142 Teff-specific probes. Red and green colors indicate high and low levels of expression, respectively. Rows correspond to individual transcripts. Columns reflect results from three individual donors denoted as 1 to 3. Cytokine clusters are highlighted by blue boxes (IL8 and IL17A at day 3; and IL3, IL4, IL9, IFNG and IL13 at day 5). Black boxes denote a cluster of inflammation-related surface proteins.

By comparing the probes regulated in iTregs and Teffs, two gene lists could be identified that contained the iTreg- and Teff-specific probes with an at least two-fold different expression between the two subsets (Fig. S1C). 93 probes corresponding to 88 transcripts were specifically up- or down-regulated in CD25+ iTregs at least at one time point during the differentiation culture. In CD25+ Teffs, 142 probes (130 transcripts) were specifically regulated. The number of the specifically regulated genes within either CD25+ T cell population gradually increased during differentiation resulting in a maximum number of genes distinguishing iTregs and Teffs at day 10 (Fig. 3A). This indicates that the specific genetic program for iTregs and Teffs, respectively, became more prominent in mature cells. Hierarchical clustering visualized distinct gene clusters characterizing both populations at every analyzed time point (Fig. 3B). For example, two cytokine clusters (IL-8 and IL-17A; and IL-3, IL-4, IL-9, IFNγ and IL-13) were up-regulated at days 3 and 5 in Teffs but not in iTregs. At days 5, 7 and 10, a gene cluster consisting of inflammation-related surface molecules such as the IL-12 receptor β2 chain (IL-12RB2) and the CC chemokine receptor 2 (CCR2) was highly expressed in Teffs but not in iTregs. Expression of several representative transcripts was validated by real time PCR (Fig. S2).

### Biological process analysis

To gain insight into the function of the regulated transcripts, we performed a biological process analysis by use of the Gene Ontology (GO) database (http://www.geneontology.org) (Fig. S1C). The over-represented processes in developing iTregs and Teffs could be grouped into three major categories all of which are related to cell cycle progression: cell cycle regulation, DNA/RNA metabolism and cytoskeleton reorganization (Table 1). This pattern supports the assumption that T cell differentiation is dependent on and associated with cell proliferation. The details of biological processes differed in iTregs and Teffs, however. Whereas the number of GO terms associated with cell proliferation decreased in developing iTregs and were absent at day 10, they remained at a largely steady level in Teffs (Table 1). This is in line with the phenotype of functionally mature iTregs, which are hypoproliferative and anergic, and Teffs, which are vigorously proliferative upon stimulation.

**Table 1:** Number of significantly over-represented biological processes within different functional categories.

Category	iTregs				Teffs			
	day 3	day 5	day 7	day 10	day 3	day 5	day 7	day 10
Cell cycle regulation	22	10	13	0	32	23	29	27
DNA/RNA metabolism	23	18	15	0	26	13	16	11
Cytoskeleton reorganization	1	0	0	0	4	2	3	3
Miscellaneous*	2	4	0	1	0	2	4	0

*Miscellaneous biological processes. iTregs: day 3 - mitochondrion organization (GO:0007005), protein targeting (GO:0006605); day 5 - NLS-bearing substrate import into nucleus (GO:0006607), regulation of transcriptional preinitiation complex assembly (GO:0045898), negative regulation of transcriptional preinitiation complex assembly (GO:0017055), gene expression (GO:0010467); day 10 - positive regulation of I-kappaB kinase/NF-kappaB cascade (GO:0043122). Teffs: day 5 - antigen processing and presentation of peptide antigen via MHC class I (GO:0002474), gene expression (GO:0010467); day 7 - sulfur metabolic process (GO:0006790), cellular amino acid metabolic process (GO:0006520), sulfur compound biosynthetic process (GO:0044272), glutation biosynthetic process (GO:0006750).

**Figure 4:**
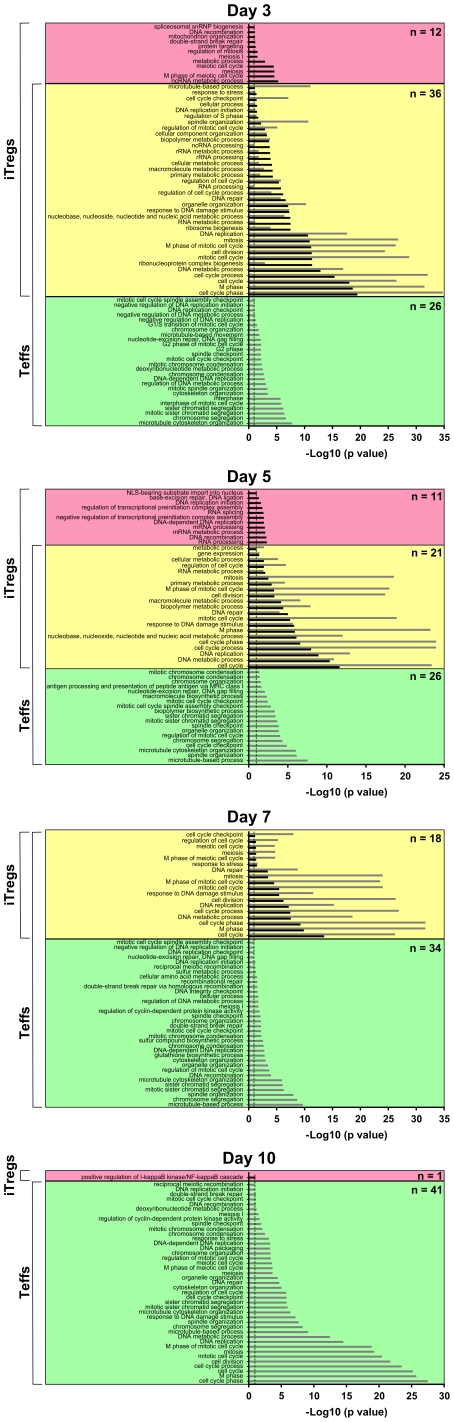
GO term enrichment analysis of biological processes in developing iTregs and Teffs. GO term analysis was performed to identify biological processes significantly over-represented in iTregs and Teffs. Enriched processes in iTregs (black bars) and Teffs (grey bars) at different times organized by their significance score (-log10 p value) are shown. Red and green boxes indicate unique GO terms for iTregs and Teffs, respectively. Yellow boxes represent GO terms enriched in the gene lists of both, iTregs and Teffs.

Early during differentiation, some of these processes were specifically represented in iTregs or in Teffs. The majority of biological processes were, however, utilized by both cell populations (Fig. 4, Fig. S3). During differentiation, the number of overlapping processes decreased, and in mature iTregs and Teffs active biological processes were mutually exclusive. The single GO term significantly over-represented in mature iTregs was “positive regulation of I-kappaB kinase/NF-kappaB cascade” (GO:0043122) comprising IL1 beta (39402_at, Hs.126256), IL1 receptor like 1 (207526_s_at, Hs.66), lymphotoxin alpha (206975_at, Hs.36), tetraspanin 6 (209108_at, Hs.43233), extracellular matrix protein 1 (209365_s_at, Hs.81071), neurotrophic tyrosine kinase receptor type 1 (208605_s_at, Hs.406293), receptor-interacting serin-threonin kinase 2 (209545_s_at, Hs.103755), CASP8 and FADD like apoptosis regulator (210563_x_at, Hs.390736), and gap junction protein alpha 1 (201667_at, Hs.74471).

### Pathway analysis

**Figure 5:**
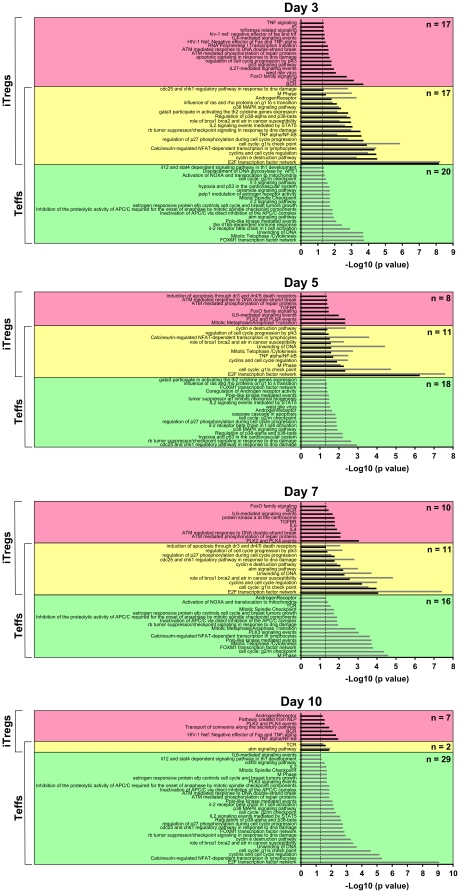
Pathway analysis in developing iTregs and Teffs. Pathway analysis was performed to identify biological pathways significantly over-represented in iTregs and Teffs. Enriched processes in iTregs (black bars) and Teffs (grey bars) at different times organized by their significance score (-log10 p value) are shown. Red and green boxes indicate unique pathways for iTregs and Teffs, respectively. Yellow boxes represent pathways enriched in the gene lists of both, iTregs and Teffs.

**Figure 6:**
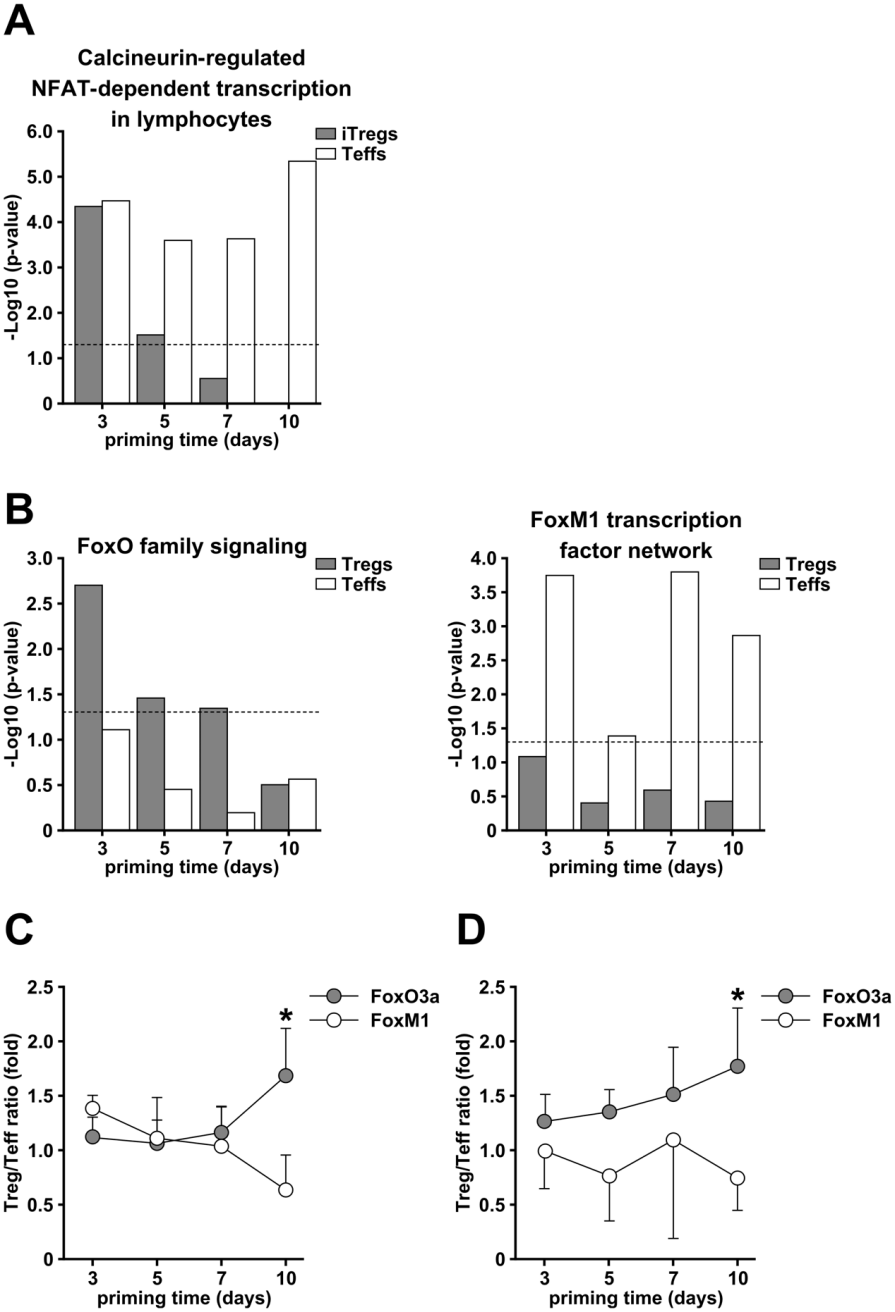
Pathways characteristic for developing iTregs or Teffs. (A, B) Significance score (-log10 p value) of three pathways characteristic for iTregs or Teffs development, “calcineurin-regulated NFAT-dependent transcription in lymphocytes” (A), “FoxO family signaling” and “FoxM1 transcription network” (B) during the 10 days of culture. Grey bars indicate significance scores in iTregs, white bars in Teffs. (C, D) Relative expression of the transcription factors FoxO3a and FoxM1 as a ratio of their mRNA expression levels in iTregs to those in Teffs at each respective time point determined by microarray (C) and by real-time PCR (D). Data are shown as mean ± SD from three (C) and five (D) experiments. * p<0.05.

As an alternative analysis, we subjected the iTreg- and Teff-characteristic gene lists to a pathway analysis scanning immune and cancer pathways pre-loaded in GeneSpring GX10, human metabolic pathways from the KEGG database and human pathways from the Nature Pathway Interaction database (Fig. S1C). Similar to biological processes, early during the differentiation a significant proportion of pathways was utilized by both, iTregs and Teffs, whereas at day 10 a pathway dichotomy became obvious (Fig. 5). The number of significantly enriched pathways identified in iTregs gradually decreased with differentiation (34, 19, 21 and 9 pathways at days 3, 5, 7 and 10, respectively). In contrast, they remained at relatively high numbers in developing and mature Teffs (37, 29, 27 and 31 pathways at days 3, 5, 7 and 10, respectively). This pattern emphasizes once again the quiescent phenotype of mature iTregs as compared to T effector cells. Consistent with this, a pathway characteristic for T cell activation, the calcineurin-regulated NFAT-dependent transcription pathway, was significantly represented in iTregs only at days 3 and 5, whereas it was active throughout the culture in Teffs (Fig. 6A). No pathways consisting exclusively of down-regulated transcripts could be identified in iTregs suggesting that developing iTregs do not repress a particular pathway (Table S1).

Further analysis of pathways specifically represented in iTregs or in Teffs identified a reciprocal pattern of two signaling pathways of Fox family transcription factors, FoxO and FoxM1 (Fig. 6B). The schematic illustration of FoxO and FoxM1 transcription factor networks and the expression profile of transcripts regulated in iTregs and Teffs are shown in Figs. S4 and S5. Analysis of the expression profile revealed an up-regulation of FoxO3a (204132_s_at) in iTregs at days 7 and 10, whereas FoxM1 (202580_x_at) was up-regulated in Teffs, resulting in a shift of the FoxO3a/FoxM1 ratio in mature populations (Fig. 6C). This divergent expression pattern was confirmed by real-time PCR analysis in independent experiments (Fig. 6D). These results suggest a potential decisive role of the Fox transcription factor family to determine iTreg versus Teff development during T cell differentiation.

## Discussion

In this study, we analyzed the transcriptional program of developing CD25+ iTregs using DNA microarray technology. By comparing the iTreg transcriptome profile with the genes regulated in Teffs at different time points during their development we identified genes differentially expressed in iTregs compared to Teffs cells at early, middle and late stages of their maturation. Based on these gene programs, biological processes and cellular pathways overrepresented in developing cells at each particular time point could be deduced. This analysis revealed an activated and proliferative phenotype of iTregs at early developmental stages that gradually became transcriptionally quiescent during later development and at the fully differentiated stage of maturation. Importantly, mature iTregs demonstrated the highest transcriptional diversity with activated Teffs. The iTreg and Teff transcriptome profiling suggested FoxO family and FoxM1 transcription factor pathways as decisive molecular mechanisms regulating iTreg development and activation of Teffs, respectively.

To our knowledge, this is the first study that analyzed the global transcriptome profile and molecular program of human iTreg development at different developmental stages. Different stimuli have been described that drive iTreg conversion from CD25- precursors in the periphery *in vivo* and *in vitro*, such as subimmunostimulatory antigen presentation *in vivo* [30], [31], TGFβ [32], [33], retinoic acid [34]-[37], IFNγ [38], [39] or particular neuropeptides (reviewed in [22]). However, a systematic analysis of molecular processes that facilitate iTreg development in the periphery has not been carried out. We took advantage of a previously described *in vitro* cell culture system which allows iTreg generation from human naive CD4 CD25- T cells over 10 days in response to suboptimal stimulation with autologous antigen-presenting cells and IL-4 [23]. CD25+ iTregs generated in this setting phenotypically and functionally resemble nTregs, as they express CD25 and Foxp3, are anergic to mitogenic stimulation and inhibit the proliferation of effector T cells in response to CD3 stimulation [23]. Therefore, analysis of the gene expression profile of those CD25+ iTregs at different time points was thought to allow the identification of genes specifically regulated in Tregs compared to Teffs and provide insight into the mechanisms underlying Treg cell development in the periphery.

CD25 and the transcription factor Foxp3 are the best described and mostly utilized markers for CD25+ Tregs [4], [40]. Beside CD25 and Foxp3, a large proportion of genes reported as Treg-specific genes in nTregs and in TGFβ-induced iTregs [27], [41], [42] were similarly regulated in mature iTregs generated in our system. For example, IL2RB, CTLA4, ICOS, IL1R1, IL1RL1, LAG3, CD103, TRAF1, OX40, CD86, LGALS1, TNFRSF9, TNFRSF1B, MAF, IRF4, SOCS2, KLRG1, DUSP4 were up-regulated in our analysis (Table S2) and in nTregs and in TGFβ-induced iTregs [27], [41], [42]. On the other hand, IL7R, NELL2, CCR7, ID2, IFNG were down-regulated here (Table S2) as they were in the previous reports. As the HG_U133A microarray platform does not include probes for GITR, FOLR4, and GRP83, the expression of these genes could not be analyzed. Nevertheless, GITR expression was confirmed in IL-4 induced iTregs by surface staining [23]. A different surface marker, PECAM1, reported to be specific for nTregs [43], was down-regulated in our iTregs. This may be related to their induced nature, since only naive nTreg cells have been shown to express PECAM1 [43].

With respect to cytokine production, Treg cells are deficient in the production of pro-inflammatory cytokines [2], [44]. In our system, we observed increased IFNG and IL17A gene expression in Teffs but not in early and late developing iTregs (Fig. 3B). Another cluster of Th2-characteristic cytokine genes, IL4, IL13, IL3 and IL9, showed enhanced expression in Teffs but not in iTregs at day 5 (Fig. 3B), supporting the conclusion of a compromised ability of maturating and mature Tregs to produce effector cytokines in contrast to Teffs [2], [44]. Moreover, when comparing mature iTregs and Teffs (day 10), down-regulation of a number of cytokine genes such as IFNG, IL2, IL1A, IL8 and IL21 was determined in iTregs. The gene expression pattern of Teffs was at large comparable with published data sets of effector T cells [27], [42] (Table S3). Of interest, Foxp3 was regulated during the development of Teffs in our system (Fig. 1B). Foxp3 is expressed in human T cells upon activation without mediating a regulatory capacity [45]. Our finding, therefore, is not surprising and in line with previous reports.

A model of early development of different T cell subsets including effector and regulatory T cells in the periphery has been proposed that is characterized by an activation-specific overlapping expression pattern [46]. Accordingly, at later stages, Treg lineage-specific regulatory genes (such as Foxp3) reduce the spectrum of effector genes in Tregs, thereby defining their regulatory functions. Indeed, our analysis of genes regulated in iTregs and in Teffs at different time points revealed a strong overlap at the beginning of the development with increasing differences between expression profiles of developing iTregs and Teffs up to the most pronounced differences between mature iTregs and Teffs. The increasing numbers of iTreg-specific and Teff-specific transcripts throughout the culture time from day 3 to day 10 illustrate this phenomenon (Fig. 3A). These results correlate with markedly different functional phenotypes of mature iTregs and activated Teffs [47].

Increasing differences between iTregs and Teffs during maturation were also obvious when the functional relevance of the iTreg-regulated and Teff-regulated transcripts was analyzed by “pGO enrichment and pathway analysis. Similar to transcripts, biological processes and pathways that were overrepresented in developing iTregs decreased in numbers from early to late developmental stages. In Teffs, in contrast, the numbers of enriched GO terms and pathways remained high during the whole culture. This suggests a declining cellular activity of iTregs during their maturation contrary to constantly activated effector T cells. The biological processes enriched within maturating iTreg- and Teff-regulated genes from day 3 to day 7 were common and predominantly represented processes controlling proliferation and cell cycle progression. In mature iTregs, however, the transcripts expressed did not statistically fit the proliferative process construct, together indicating a vigorous proliferative status of immature iTregs followed by a decreasing proliferative capacity during maturation and an anergic phenotype of mature iTregs. In this regard, a decline of the proliferative capacity of maturating iTregs in response to anti-CD3 stimulation was observed from day 7 to 14 of the culture (data not shown). Although overrepresented in both populations, biological processes regulating proliferation reached a higher significance level in Teffs than in iTregs, indicating less active proliferation of developing iTregs as compared to Teffs. This is in line with a previously published report of an inverse relationship between cell division and Treg conversion, i.e. limited proliferation is a requirement for effective peripheral Treg conversion [30].

Foxp3 is a “master regulator“ for Treg development [48], [49]. Accumulating evidence suggests that Foxp3 functions within a higher-order regulation network of signaling molecules and transcription factors during Treg establishment [27]. Deprived TCR signaling via inhibition of the PI3K/Akt signaling pathway by rapamycin (mTOR) induces *de novo* expression of Foxp3 and Treg-like gene expression profiles [50]. Similarly, Akt has been identified as a strong repressor of entry into the Treg phenotype *in vitro* and *in vivo* [51]. Notably, the activation of the PI3K/Akt pathway is impaired in Tregs as indicated by a reduction in Akt phosphorylation in these cells [52]. However, the molecular links between impaired PI3K/Akt signaling and subsequent FoxP3 expression are not completely understood. Here, we identified the “FoxO family signaling” pathway as significantly represented in iTregs during maturation. The expression of FoxO3a, one of the key transcription factors of the pathway, was increased in iTregs compared to Teffs during maturation (Fig. 6). Activity of FoxO3a is directly regulated by Akt, which phosphorylates FoxO3a resulting in the inactivation of its function by exclusion from the nucleus [53]. Thus, suboptimal TCR stimulation in combination with an inhibition of PI3K/Akt signaling might result in FoxO3a accumulation in the nucleus of iTregs and participate in the induction of Foxp3 expression [54], [55]. Indeed, phosphorylation of FoxO3a is reduced in Treg cells [52]. Dephosphorylated FoxO3a regulates, moreover, the transcription of genes promoting cell cycle arrest [56] that could support its role in restricting the proliferation of iTregs during their maturation period. Of note, a key component of TGFβ-mediated inhibition of cell proliferation is a formation of a Smad-FoxO3a complex [57], suggesting a central role of FoxO signaling in Treg development in the periphery.

FoxO3a is also known to repress FoxM1 expression and activation [58], [59]. FoxM1 has a critical role in cell proliferation by regulating various cell cycle regulatory genes [59]. Loss of FoxM1 is associated with mitosis arrest and disrupted mitotic spindle integrity [60]. In this study, the “FoxM1 transcription factor network pathway” was significantly represented in developing and mature Teffs. The representation of this pathway correlates with their highly proliferative phenotype throughout development. In contrast, expression of FoxM1 in Tregs declined from early to late maturation stages, together suggesting an intriguing link between FoxO3a and FoxM1 transcription factor signaling in determining the fate of a Treg or Teff.

In conclusion, using microarray analysis of the transcriptional program of developing iTregs we provide new insight into Treg development in the periphery. The detailed understanding of molecules and pathways involved in peripheral Treg differentiation might provide new therapeutic targets for the treatment of disorders caused by dysregulation of Tregs, such as autoimmune diseases, chronic infection and graft rejection.

## Supporting Information

Figure S1 **Microarray gene analysis of iTreg development.** (A) Schematic drawing of the experimental strategy. iTregs and Teffs were generated *in vitro* from naive CD25- CD4 T cells by stimulation with autologous feeder cells in the presence or absence of IL-4, respectively. Total RNA was isolated from CD25- CD4 T cells before culture (day 0) and from purified CD25+ and CD25- subsets at days 3, 5, 7 and 10 of the cultures and hybridized on HG_U133A microarray chips. Data sets (n = 50) from three independent experiments with cells from different donors were subjected to baseline transformation and the probes were filtered by flags resulting in a gene list of 15,245 probes with a marginal or present call in at least one of the 50 samples. (B) Strategy of the statistical analysis. In the first step, one-way ANOVA was performed to determine those genes that were not regulated in CD25- cells throughout the culture assuming that the changes in gene expression in CD25- cells during the culture reflected unspecific cell culture interference. The second step was designed as a two-way ANOVA followed by fold-change analysis testing to identify probes that were at least two-fold up- or down-regulated in Teffs or iTregs compared to the corresponding CD25- T cells at each culture time point. (C) Identification of specific genes and characteristic biological processes and pathways. The gene lists from Step 2 were further processed by alternative statistical analyses: step 3A employed one-way ANOVA to identify genes that were specifically regulated in iTregs and Teffs; step 3B performed a GO term analysis of biological processes over-represented in developing iTregs and Teffs; step 3C utilized the Cancer Cell Map, BioCyc, KEGG and Nature Pathway Interaction databases to conduct a pathway analysis in developing iTregs and Teffs.
(TIF)

Figure S2 **Real time PCR analysis.** Expression of MAFF (Hs.517617), CCR2 (Hs.644637), PGDS (Hs.128433), and PMCH (Hs.707990) in developing iTregs and Teffs from gene chip (A) and from real time PCR analysis (B). Data are shown as mean+SD from three (A) and eight (B) experiments in relation to the expression in naive T cells (day 0). * p<0.05.
(TIF)

Figure S3 **Hierarchical network organization of the significantly enriched GO terms.** Each box represents one GO term and the p value of its enrichment. p1 and p2 values correspond to the p values in iTregs and Teffs, respectively. Red and green boxes indicate unique GO terms for iTregs and Teffs, respectively. Yellow boxes represent GO terms enriched in the gene lists of both, iTregs and Teffs.
(TIF)

Figure S4 **Network organization of “FoxO family signaling” and “FoxM1 transcription factor network” pathways.** Each node represents proteins, small molecules or protein complexes participating in the pathway. Nodes marked by black cycles correspond to the transcripts identified by the microarray as to be regulated in developing iTregs (“FoxO family signaling”) or in developing Teffs (“FoxM1 transcription factor network”).
(TIF)

Figure S5 **FoxO and FoxM1 pathways.** Expression of the specific transcripts from “FoxO family signaling” and from “FoxM1 transcription factor network” pathways determined by microarray analysis is shown as mean+SD from results of three donors.
(TIF)

Table S1 (XLS)

Table S2 (DOC)

Table S3 (DOC)
