# Supplementary figures and images for: Analysis of the Transcriptional Program of Developing Induced Regulatory T Cells
Source: PLoS One. 2011 Feb 9;6(2):e16913. doi: 10.1371/journal.pone.0016913 (PMC3036712; doi:10.1371/journal.pone.0016913)

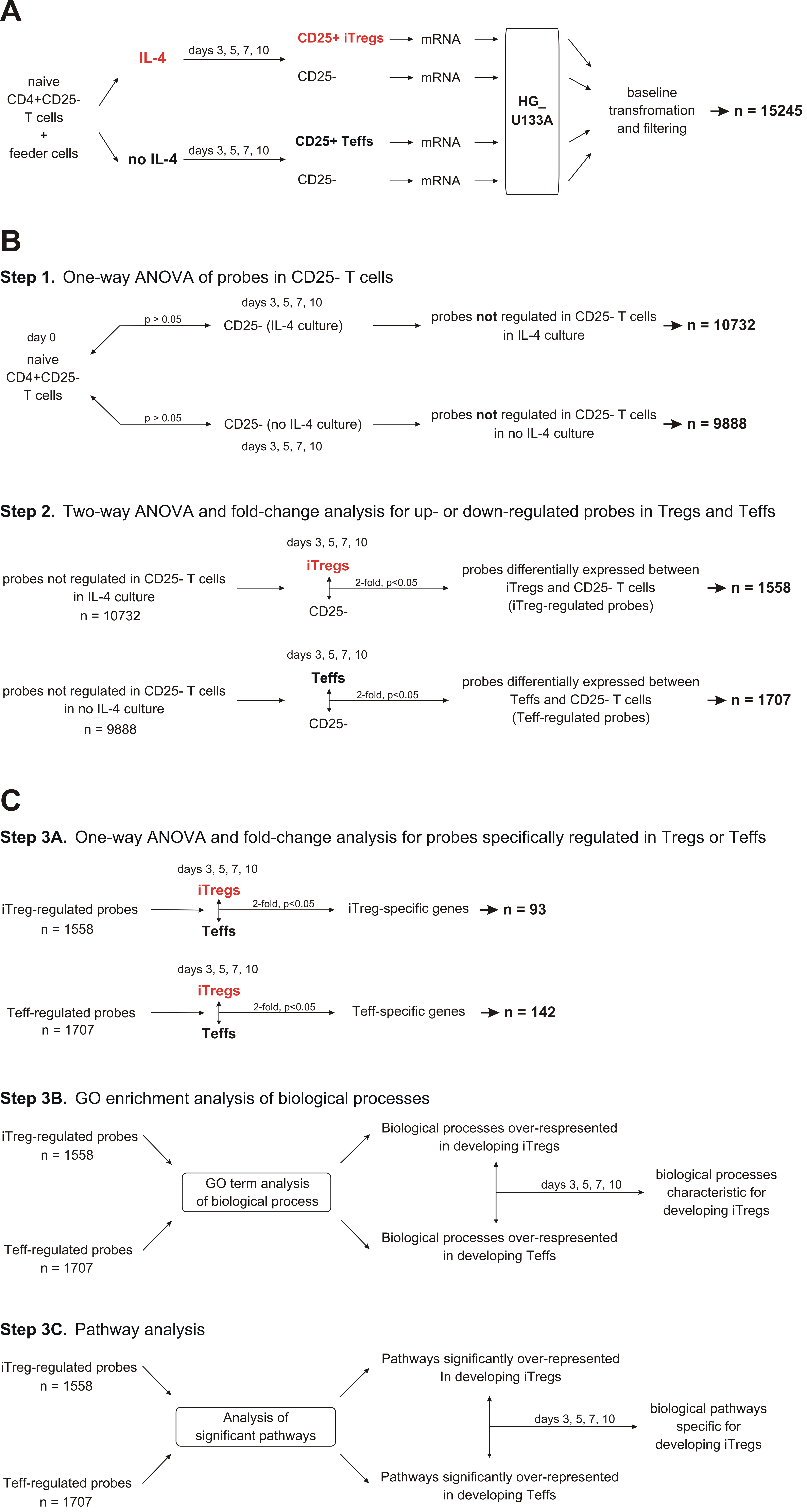

Supplement: Figure S1 — Microarray gene analysis of iTreg development. (A) Schematic drawing of the experimental strategy. iTregs and Teffs were generated in vitro from naive CD25- CD4 T cells by stimulation with autologous feeder cells in the presence or absence of IL-4, respectively. Total RNA was isolated from CD25- CD4 T cells before culture (day 0) and from purified CD25+ and CD25- subsets at days 3, 5, 7 and 10 of the cultures and hybridized on HG_U133A microarray chips. Data sets (n = 50) from three independent experiments with cells from different donors were subjected to baseline transformation and the probes were filtered by flags resulting in a gene list of 15,245 probes with a marginal or present call in at least one of the 50 samples. (B) Strategy of the statistical analysis. In the first step, one-way ANOVA was performed to determine those genes that were not regulated in CD25- cells throughout the culture assuming that the changes in gene expression in CD25- cells during the culture reflected unspecific cell culture interference. The second step was designed as a two-way ANOVA followed by fold-change analysis testing to identify probes that were at least two-fold up- or down-regulated in Teffs or iTregs compared to the corresponding CD25- T cells at each culture time point. (C) Identification of specific genes and characteristic biological processes and pathways. The gene lists from Step 2 were further processed by alternative statistical analyses: step 3A employed one-way ANOVA to identify genes that were specifically regulated in iTregs and Teffs; step 3B performed a GO term analysis of biological processes over-represented in developing iTregs and Teffs; step 3C utilized the Cancer Cell Map, BioCyc, KEGG and Nature Pathway Interaction databases to conduct a pathway analysis in developing iTregs and Teffs. (TIF) [file pone.0016913.s001.tif]

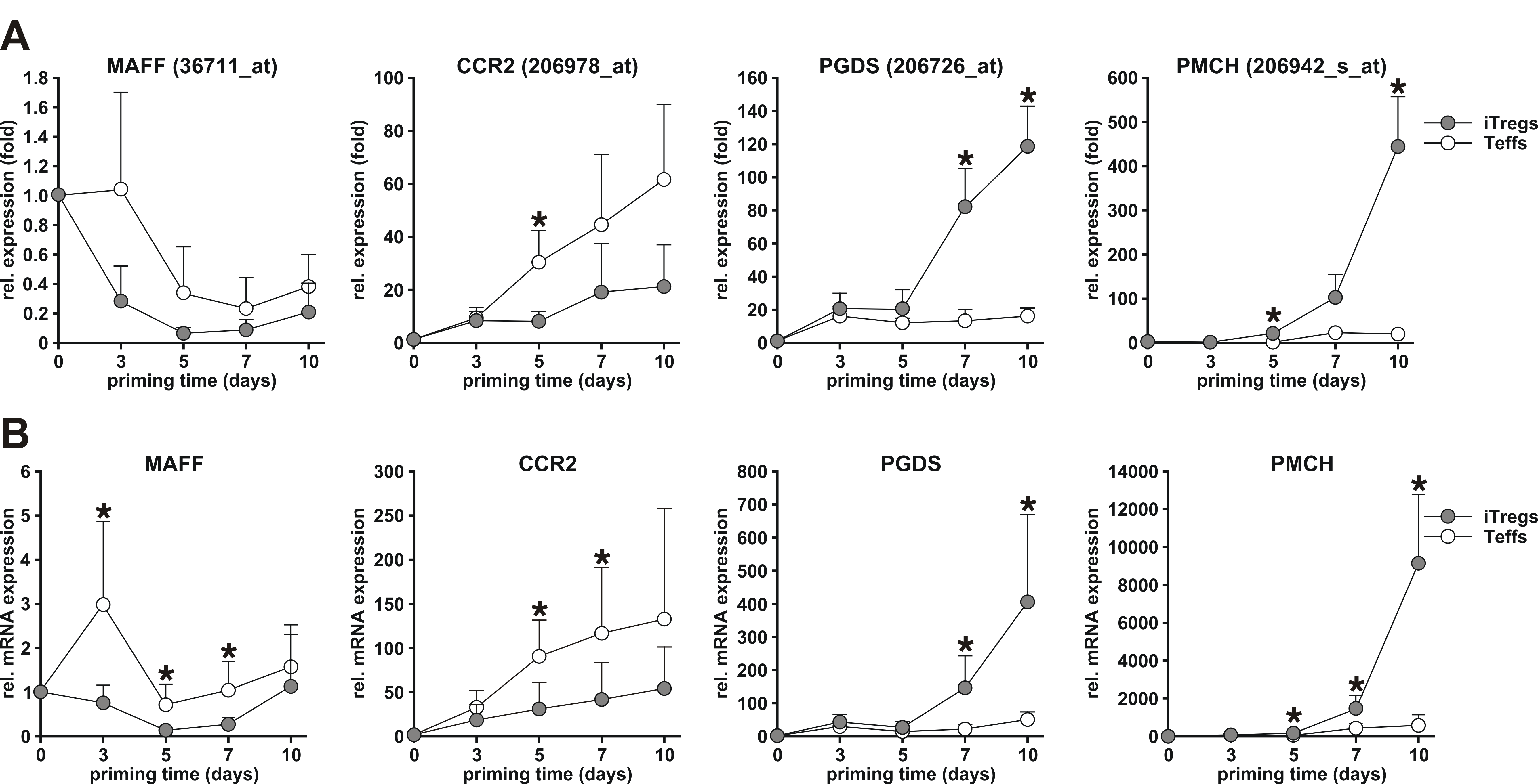

Supplement: Figure S2 — Real time PCR analysis. Expression of MAFF (Hs.517617), CCR2 (Hs.644637), PGDS (Hs.128433), and PMCH (Hs.707990) in developing iTregs and Teffs from gene chip (A) and from real time PCR analysis (B). Data are shown as mean+SD from three (A) and eight (B) experiments in relation to the expression in naive T cells (day 0). * p<0.05. (TIF) [file pone.0016913.s002.tif]

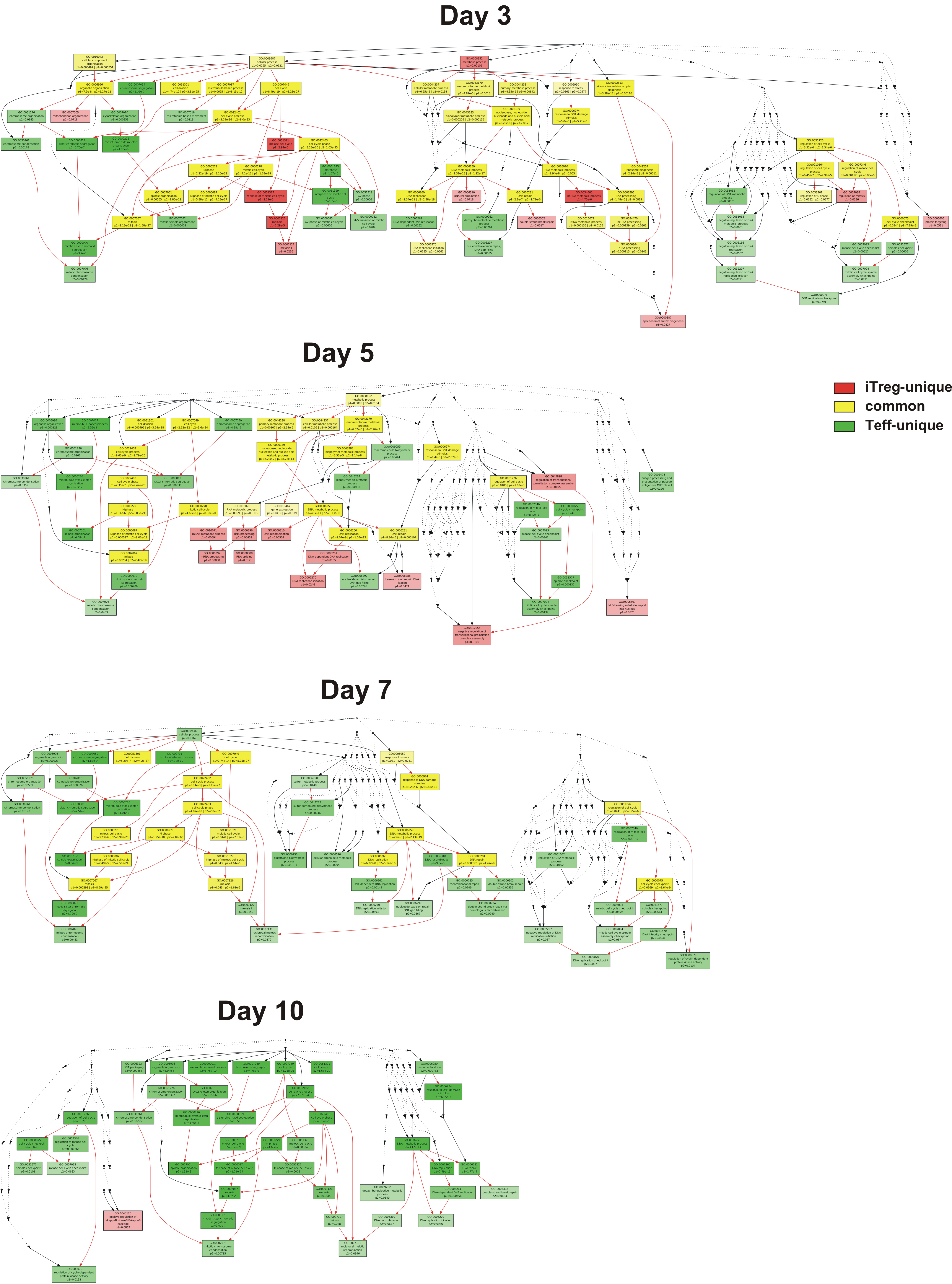

Supplement: Figure S3 — Hierarchical network organization of the significantly enriched GO terms. Each box represents one GO term and the p value of its enrichment. p1 and p2 values correspond to the p values in iTregs and Teffs, respectively. Red and green boxes indicate unique GO terms for iTregs and Teffs, respectively. Yellow boxes represent GO terms enriched in the gene lists of both, iTregs and Teffs. (TIF) [file pone.0016913.s003.tif]

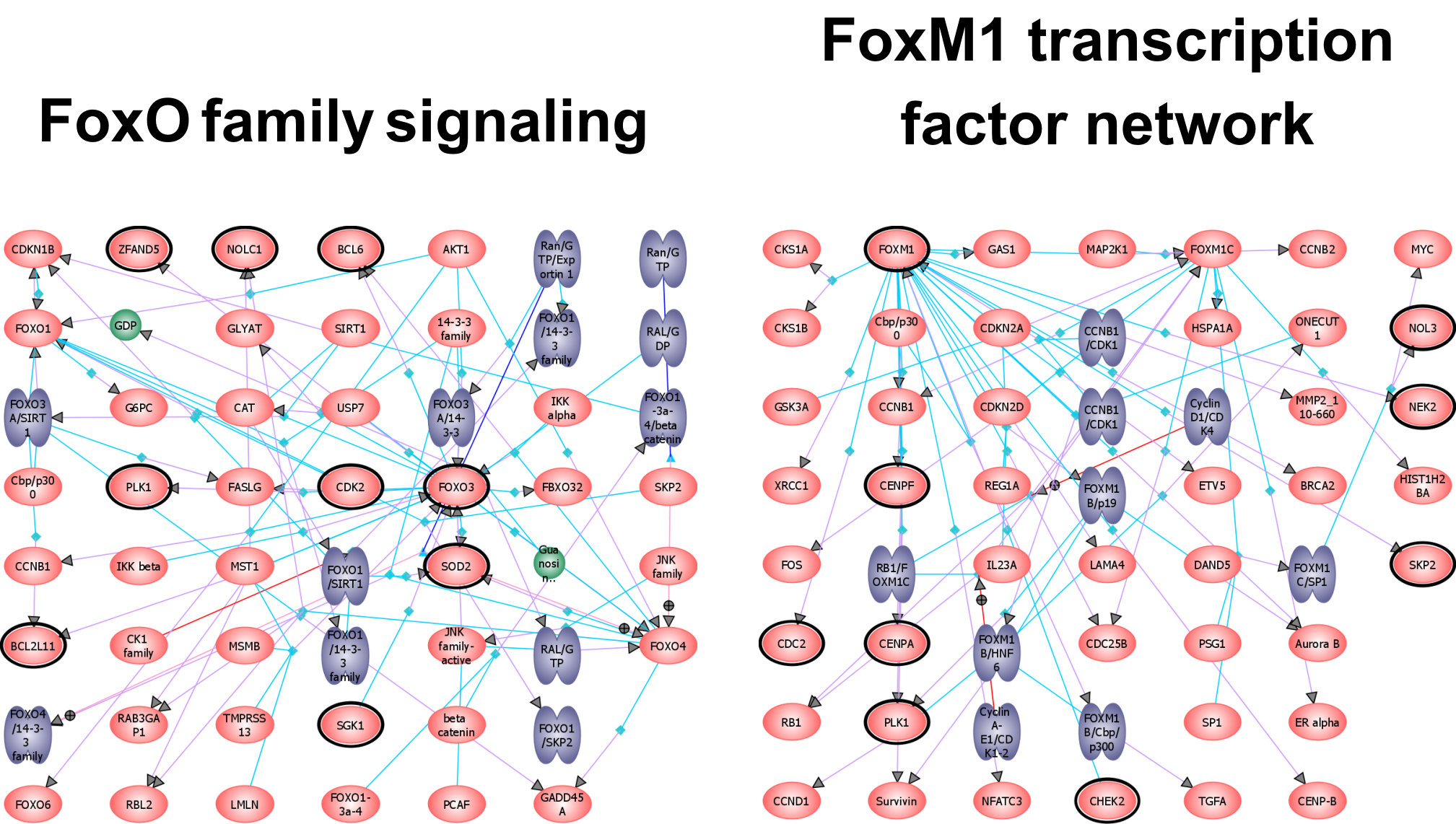

Supplement: Figure S4 — Network organization of “FoxO family signaling” and “FoxM1 transcription factor network” pathways. Each node represents proteins, small molecules or protein complexes participating in the pathway. Nodes marked by black cycles correspond to the transcripts identified by the microarray as to be regulated in developing iTregs (“FoxO family signaling”) or in developing Teffs (“FoxM1 transcription factor network”). (TIF) [file pone.0016913.s004.tif]

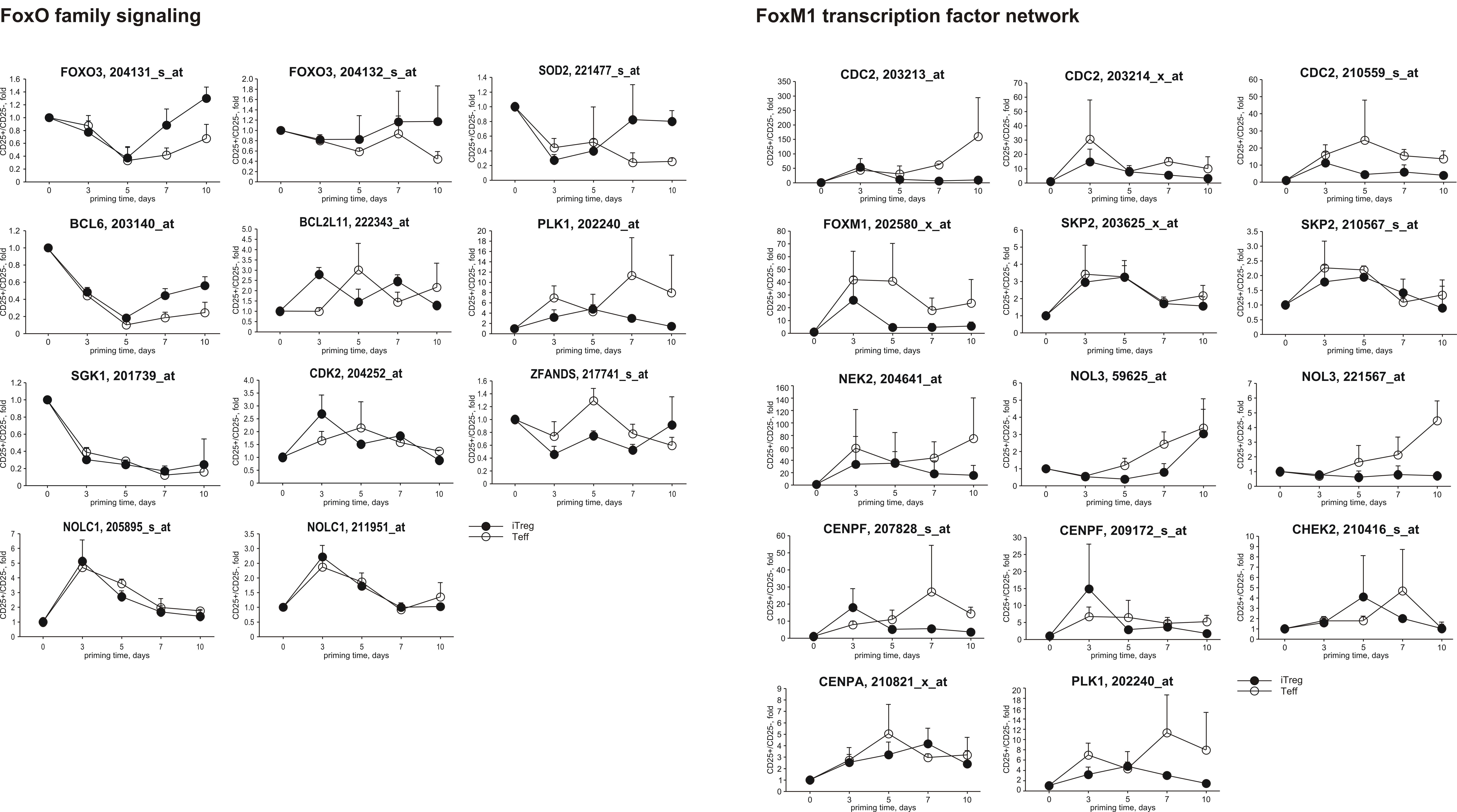

Supplement: Figure S5 — FoxO and FoxM1 pathways. Expression of the specific transcripts from “FoxO family signaling” and from “FoxM1 transcription factor network” pathways determined by microarray analysis is shown as mean+SD from results of three donors. (TIF) [file pone.0016913.s005.tif]
