# Supplementary material for: Analysis of the Transcriptional Program of Developing Induced Regulatory T Cells
Source: PLoS One. 2011 Feb 9;6(2):e16913. doi: 10.1371/journal.pone.0016913 (PMC3036712; doi:10.1371/journal.pone.0016913)
Supplement: Table S2 — (DOC) [file pone.0016913.s007.doc]

Table S2. Common regulatory T cell-characteristic transcripts.*

| **Transcript (probe)** | **Tregs/naive CD4 CD25- T** | |
| --- | --- | --- |
|  | **fold change** | **p value** |
| Surface molecules: |  |  |
| IL2RB (205291_at) | 2.4 | 0.0151 |
| CTLA4 (221331_x_at) | 14.8 | 0.0049 |
| CD103 (205055_at) | 2.4 | 0.0014 |
| IL1R1 (202948_at) | 3.0 | 0.0043 |
| IL1RL1 (207526_s_at) | 5.3 | 0.0382 |
| LAG3 (206486_at) | 5.1 | 0.0261 |
| CD86 (210895_s_at) | 9.6 | 0.0018 |
| CD86 (205685_at) | 7.1 | 0.0139 |
| CD86 (205686_s_at) | 7.3 | 0.0247 |
| OX40/TNFRSF4 (214228_x_at) | 13.4 | 0.0076 |
| TNFRSF9 (207536_s_at) | 2.7 | 0.0036 |
| TNFRSF1B (203508_at) | 4.5 | 0.0037 |
| KLRG1 (210288_at) | 2.1 | 0.0066 |
| ICOS (210439_at) | 1.8 | >0.050 |
| IL7R (205798_at) | - 2.1 | 0.0149 |
| CCR7 (206337_at) | - 2.0 | 0.0163 |
| PECAM1 (208981_at) | - 1.6 | 0.0368 |
| Signal transduction: |  |  |
| TRAF1 (205599_at) | 2.1 | 0.0005 |
| SOCS2 (203373_at) | 2.2 | 0.0325 |
| IRF4 (204562_at) | 2.5 | 0.0045 |
| MAF (209348_s_at) | 5.7 | 0.0043 |
| DUSP4 (204014_at) | 11.4 | 0.0034 |
| DUSP4 (204015_s_at) | 9.3 | 0.0426 |
| NELL2 (203413_at) | - 4.9 | 0.0008 |
| ID2 (201565_s_at) | - 1.5 | 0.0415 |
| Soluble factors: |  |  |
| LGALS1 (201105_at) | 12.0 | 0.0058 |
| GZMA (205488_at) | 2.7 | 0.0415 |
| IFNG (210354_at) | - 8.2 | 0.0500 |

*Transcripts were filtered based on differential expression (fold change at least 1.5, p ≤ 0.05) in Tregs (day 10) in comparison to naive CD4 CD25- T cells (day 0). The analysis of expression was performed using GeneSpring GX10. Transcripts are shown if regulated in this study and in nTregs or TGF-induced iTregs [27,41,42].
