# Supplementary material for: Analysis of the Transcriptional Program of Developing Induced Regulatory T Cells
Source: PLoS One. 2011 Feb 9;6(2):e16913. doi: 10.1371/journal.pone.0016913 (PMC3036712; doi:10.1371/journal.pone.0016913)
Supplement: Table S3 — (DOC) [file pone.0016913.s008.doc]

Table S3. Common effector T cell-characteristic transcripts.*

| **Transcript (probe)** | **Teffs/naive CD4 CD25- T** | |
| --- | --- | --- |
|  | **fold change** | **p value** |
| Surface molecules: |  |  |
| IL2RA (206341_at) | 29.3 | 0.0017 |
| IL2RA (211269_s_at) | 21.1 | 0.0111 |
| IL2RB (205291_at) | 3.1 | 0.0325 |
| ICOS (210439_at) | 2.8 | 0.0261 |
| CTLA4 (221331_x_at) | 26.3 | 0.0112 |
| ICAM1 (202637_s_at) | 6.5 | 0.0082 |
| ICAM1 (202638_s_at) | 3.3 | 0.0420 |
| CD86 (205685_at) | 16.6 | 0.0185 |
| CD86 (205686_s_at) | 15.2 | 0.0193 |
| CD86 (210895_s_at) | 21.4 | 0.0054 |
| HLA-DRA (210982_s_at) | 74.8 | 0.0001 |
| IL1R1 (202948_at) | 3.2 | 0.0060 |
| IL1R2 (205403_at) | 5.3 | 0.0372 |
| IL1R2 (211372_s_at) | 6.0 | 0.0035 |
| IL12RB2 (206999_at) | 5.2 | 0.0140 |
| IL21R (219971_at) | 2.5 | 0.0498 |
| IL13RA1 (201887_at) | 6.5 | 0.0026 |
| Signal transduction: |  |  |
| TRAF1 (205599_at) | 2.1 | 0.0042 |
| NFAT5 (215092_s_at) | 2.0 | 0.0209 |
| STAT1 (200887_s_at) | 3.9 | 0.0068 |
| MAF (209348_s_at) | 6.0 | 0.0148 |
| IRF4 (204562_at) | 2.5 | 0.0004 |
| Cytokines and soluble factors: |  |  |
| IFNG (210354_at) | 2.8 | 0.0003 |
| TNF (207113_s_at) | 2.6 | 0.0398 |
| IL15 (205992_s_at) | 3.7 | 0.0076 |
| GZMA (205488_at) | 4.7 | 0.0121 |

*Transcripts were filtered based on differential expression (fold change at least 1.5, p ≤ 0.05) in Teffs (day 10) in comparison to naive CD4 CD25- T cells (day 0). The analysis of expression was performed using GeneSpring GX10. Transcripts are shown if regulated in this study and in effector T cells previously embedded in public database [27,42].
